# The importance of being the HGNC

**DOI:** 10.1186/s40246-022-00432-w

**Published:** 2022-11-15

**Authors:** Elspeth A. Bruford, Bryony Braschi, Liora Haim-Vilmovsky, Tamsin E. M. Jones, Ruth L. Seal, Susan Tweedie

**Affiliations:** 1 Department of Haematology, University of Cambridge School of Clinical Medicine, Cambridge, CB2 0PT UK; 2 HUGO Gene Nomenclature Committee, European Molecular Biology Laboratory, EMBL-EBI, Wellcome Genome Campus, Hinxton, CB10 1SD UK

**Keywords:** Nomenclature, Genes, Genomics, Databases, HGNC

## Abstract

The HUGO Gene Nomenclature Committee (HGNC) has been providing standardized symbols and names for human genes since the late 1970s. As funding agencies change their priorities, finding financial support for critical biomedical resources such as the HGNC becomes ever more challenging. In this article, we outline the key roles the HGNC currently plays in aiding communication and the need for these activities to be maintained.

## Background

Everyone interested in genomics and genetics uses gene symbols. Sometimes they may not even realize they are using gene symbols, and sometimes they may be using their favourite “pet name” for a gene instead of the standardized nomenclature assigned by one of the established nomenclature committees, but nevertheless they are using them. Type “BRCA1” into Google and you get over 21 million results, and you can be pretty sure that the vast majority of the results returned will truly be related to the BRCA1 tumour suppressor gene. Go into PubMed and “BRCA1” brings back over twenty thousand articles. Search the BBC website with “BRCA1” and you get over 16 pages of results. Gene symbols are everywhere… and they are not going anywhere. They’re a useful shorthand way to refer to genes—while not all may be easily memorable, many researchers have memorized the symbols for their “favourite” genes (even if they don’t particularly like them!), and lots of patient support groups are named after the causative gene(s) for a particular condition, such as the DDX3X Foundation, the PCDH19 Alliance and the International FOXG1 Foundation. Furthermore, gene symbols are more and more commonly found in clinical reviews, in test results, and in patient reports, as genetics and genomics become a routine aspect of healthcare.

## Main text

So who decides what symbol a gene is “given”? For the human genome this is decided by the HUGO Gene Nomenclature Committee, or the HGNC for short (we appreciate the utility of acronyms). The HGNC has been operating for over 40 years and runs a freely accessible website at www.genenames.org listing all of the “approved” standardized gene symbols and names for human genes [1]. Each HGNC “symbol report” includes a gene symbol (usually an acronym of the gene name), a longer form descriptive gene name, a locus type stating if the gene is protein coding, a long non-coding RNA, a pseudogene, etc., and an HGNC ID which is a unique ID associated with the sequence of the gene—such that it would only ever change if the gene model also changed significantly, through merging or splitting. This means that HGNC gene IDs are not altered by changing genome assemblies, annotation runs, etc., and are likely the most stable IDs available for human genes.

HGNC also catalogs other unofficial symbols and names that have been used in the literature and databases for each gene, as well as keeping a record of previous symbols and names if these have ever been approved. Each symbol report includes the chromosomal location of the gene, and if it is a member of an HGNC gene group (more about these later). And the report provides numerous links to important and heavily used external resources, such as Ensembl [2], NCBI Gene, GenBank [3], UniProt [4], ClinGen [5], OMIM [6], GeneCards [7], and even PubMed [3] for selected key publications about the gene.

Gene nomenclature committees exist for key model organisms as well—mouse [8], rat [9], chicken [9, 10], *Xenopus* [11], zebrafish [11, 12], *C. elegans* [13], *Drosophila* [14], *S. cerevisiae* [15], *S. pombe* [16], etc., and even better, these committees regularly talk to each other, especially within the vertebrates where the majority of gene naming follows the human gene nomenclature, hence making it easy to identify orthologs and paralogs between species. You will find links to the mouse and rat orthologs of human genes in the HGNC symbol reports.

In 2016 the HGNC established a sister committee, the Vertebrate Gene Nomenclature Committee (VGNC, vertebrate.genenames.org), to ensure there is standardized gene naming in selected vertebrates not already covered by the committees mentioned above (currently chimpanzee, macaque, dog, cat, cattle, pig and horse). The VGNC project ensures that not only are genes named systematically across many vertebrate species, making it easy to identify orthologs through their shared nomenclature—and often paralogs too through a shared root symbol—but further that this nomenclature is consistent across resources, making it simple to navigate between different sites and find out as much information as possible about a given gene or set of genes. Again, links to orthologs of a human gene in a VGNC species are found in the HGNC symbol reports.

HGNC symbol reports additionally include a separate tab labelled “HCOP homology predictions”, which includes information from 14 resources on the predicted orthologs of the gene in up to 19 species. This is a snapshot of the data available from the HCOP (HGNC Comparison of Orthology Predictions) tool [17], which can be used to search for orthologs of any human gene, including links to the predicted orthologous genes and the resources making the predictions (https://www.genenames.org/tools/hcop/).

HGNC further provides a large set of over 1600 “gene groups” [18]—sets of genes grouped by homology, shared function, complex membership, etc. One gene can belong to many groups, such as the *AKAP13* gene which is in 5 groups, including the diverse “Dbl family Rho GEFs”, “Minor histocompatibility antigens” and “MicroRNA protein coding host genes”. Many groups have a dedicated “specialist advisor”, a community expert who provides advice on naming within that family.

Numerous key resources display HGNC symbols as the authoritative source of official human gene nomenclature, and these symbols facilitate text mining and mapping between multiple resources—the unique HGNC IDs and gene symbols provided by the HGNC can be reliably compared across different databases to ensure that there is no ambiguity about which gene is which. Some databases provide automated nomenclature assignment to putative genes, but these automated assignments are often either uninformative and unmemorable database identifiers that differ across different databases, or non-unique symbols assigned due to similarity to a named gene. HGNC’s review and approval processes ensure that these cases are minimized.

HGNC’s unique IDs are also used as the defining identifiers for human genes within several key databases, such as ClinGen [5] and the Alliance of Genome Resources [19]. The creation of new HGNC IDs therefore ensures that newly annotated genes are swiftly represented in these other resources. Contrary to what one might think, new genes appear with regularity—such as newly annotated long non-coding RNA genes, or protein-coding genes found in recently sequenced genomes or that have been lurking as small open reading frames in the current reference genome [20].

Another key role the HGNC plays is to correct any naming errors. Bear in mind that some genes were named several decades ago, and hence, the nomenclature assigned can occasionally turn out to be misleading. For example, the *IGJ* gene which was named originally in 1988 was renamed to *JCHAIN* to avoid the suggestion it was encoding an immunoglobulin, when in fact it encodes a peptide that links immunoglobulins together. And especially as gene symbols are increasingly being used in the clinic, it is also important to remove any potentially offensive or pejorative terms from gene nomenclature [21]—terms that may not have been considered in this context until the role of the gene product in a specific condition has been elucidated.

Gene symbol choice can sometimes have unexpected consequences. The HGNC recently updated the symbols of 27 genes that were being auto-corrected to dates in Microsoft Excel, for example, *MARCH1* (now updated to *MARCHF1*). Such an auto-correction was avoidable if users knew to format their spreadsheets in a particular way, but oftentimes users were dealing with lists of tens of thousands of genes and had no idea the auto-correction was occurring. The prevalence of this erroneous “correction” from a gene symbol to a date was such that one study found roughly twenty percent of datasets in the literature contained these errors [22]. The HGNC was able to contact the communities working on these genes and agree upon new gene symbols that would no longer be affected by this auto-formatting issue.

HGNC regularly receive user requests to update nomenclature, especially for “placeholder” symbols such as the C#orfs, KIAAs, and FAMs. The resulting new gene symbols and names enable new discoveries—for example, that the gene previously approved as *C7orf26* actually encodes a subunit of the integrator complex and hence has been renamed *INTS15* [23]—to be clearly and effectively communicated to the world at large. Curating “gene groups” has also led to informative updates to gene nomenclature: in 2020 the nomenclature of nine genes encoding human dynein chains was updated, including two genes previously assigned uninformative placeholder symbols—*C16orf71* was updated to *DNAAF8* (dynein axonemal assembly factor 8) and *C20orf194* to *DNAAF9* (dynein axonemal assembly factor 9).

Basing discussion with experts around HGNC gene groups can be an excellent way to engage with researchers in specific fields about the nomenclature of the genes that are the most important to them. HGNC gene group resources are also popular with visitors to the website and group genes in a variety of ways (based on homology, complex subunits, historical groupings); while gene groups are not currently labelled with types, this is planned for the future in combination with reviewing and improving the nomenclature of the genes within each group. One example group is the CFAP (cilia and flagella associated protein) genes which have been named based on their FAP (flagella associated protein) orthologs in the model organism *Chlamydomonas reinhardtii*. There are currently 45 approved CFAP symbols in human, and many of them are well published, with over 300 papers in PubMed using the CFAP# gene nomenclature.

While HGNC are now aiming to stabilize gene *symbols* whenever possible, gene *names* can still be adjusted to make them more functionally informative. For example, the nomenclature of the “methyltransferase like” (METTL) genes was reviewed in consultation with HGNC’s specialist advisors in 2021 and some of their names were updated to reflect that they encode active methyltransferases. Symbol updates were also made for a few genes in this group: the little used *METTL12* and *METTL21D* were updated to *CSKMT* (citrate synthase lysine methyltransferase) and *VCPKMT* (valosin containing protein lysine methyltransferase) respectively, to reflect the enzymes’ specific substrates.

Occasionally genes have a symbol alias that is overwhelmingly used. This was the case for HGNC:3942 which had the approved nomenclature *FRAP1* for “FK506 binding protein 12-rapamycin associated protein 1” from the years 2001 to 2009. The scientific community overwhelmingly referred to the gene as "mTOR", which was problematic as this stood for “mammalian target of rapamycin” so was not transferable to non-mammalian vertebrate species like chicken and zebrafish. The HGNC collaborated with the Mouse Genomic Nomenclature Committee (MGNC) in 2009 and contacted 115 researchers. After lengthy discussions, the nomenclature committees and majority of researchers agreed upon “*MTOR*” which now stands for “mechanistic target of rapamycin kinase”. This gene symbol is well supported but has also allowed other genes to be named relative to the *MTOR* symbol, such as *DEPTOR* (DEP domain containing MTOR interacting protein), *LAMTOR1* (late endosomal/lysosomal adaptor, MAPK and MTOR activator 1), *RPTOR* (regulatory associated protein of MTOR complex 1) and *RICTOR* (RPTOR independent companion of MTOR complex 2). None of this would have been possible if the gene had languished as *FRAP1*.

The HGNC regularly deals with nomenclature queries from the clinical and scientific communities. Some of these queries are initially directed to other resources who then forward them to the HGNC—which means that even if a research group is not originally aware of the HGNC, they are nonetheless the endpoint for these queries. HGNC’s position as the worldwide human gene nomenclature authority means that they are well placed to mediate disputes between rival groups and encourage discussions to reach a consensus nomenclature for use in publications going forward, as well as disseminate any nomenclature updates to other crucial biological resources. In this role, HGNC aims to reduce duplication of effort between different research groups as well as in other biological databases and reduce the confusion that might otherwise arise when different names are used for the same gene, or the same symbols are used for different genes.

## Conclusion

Funding for the HGNC and other databases supporting scientific research has recently become precarious—Wellcome in the UK no longer supports resources like ours and NIH money is tighter too. If HGNC does not secure future funding will it matter? Is our dictionary for the human genome ever going to be sufficiently complete that it can be left untended?

With no HGNC, others may create a new “official” nomenclature—competing efforts or radical changes would result in chaos in human genetics, vertebrates and beyond. Without liaison between nomenclature committees, which is routinely instigated by HGNC, orthologous and paralogous genes would risk being named inconsistently across vertebrate model organisms, hence losing information and requiring each user to work out the relationships between genes. Journals would be unable to recommend the use of official gene symbols, especially if new symbols couldn’t be requested, or queries responded to, resulting in a free-for-all on alternative symbols for existing genes. There would be more persistent use of common English words being used as gene symbols, which would hamper literature searching and text mining efforts—we know this for a fact as we regularly receive requests to approve widely-used words as gene symbols! The use of alternative nomenclature for the same gene can result in potential waste of funding and resources: our cataloguing of aliases allows disambiguation of genes in publications. Most critically, confusion between genes in the literature and also in laboratories and clinics would rise, potentially resulting in patient harm [24]. With the advent of personalized medicine, where clinicians, genetic counsellors, patients, and their families are now talking about gene symbols, we truly believe that the role of the HGNC is needed as much now as it ever has been.
